# Targeting Cancer Translational Plasticity: IRES-Driven Metabolism and Survival Within the Tumor Microenvironment

**DOI:** 10.3390/cancers17172731

**Published:** 2025-08-22

**Authors:** Fabrizio Damiano, Benedetta Di Chiara Stanca, Laura Giannotti, Eleonora Stanca, Angela Francesca Dinoi, Luisa Siculella

**Affiliations:** 1 Department of Experimental Medicine, University of Salento, 73100 Lecce, Italy; benedetta.dichiara@unisalento.it (B.D.C.S.); laura.giannotti@unisalento.it (L.G.); eleonora.stanca@unisalento.it (E.S.); angelafrancesca.dinoi@unisalento.it (A.F.D.); luisa.siculella@unisalento.it (L.S.); 2 Institute of Polymers, Composites and Biomaterials, National Research Council of Italy (IPCB-CNR), 80078 Naples, Italy

**Keywords:** IRES-mediated translation, Warburg effect, apoptosis resistance, angiogenesis, hypoxia, metabolic reprogramming, cellular plasticity

## Abstract

The tumor microenvironment creates strong stress conditions, including hypoxia and nutrient depletion, which cause the blocking of cap-dependent translation. Under stressful conditions, cancer cells exploit the cap-independent translation mechanism mediated by internal ribosome entry site (IRES), which ensures continued protein synthesis. IRES elements located in the 5′ untranslated regions of specific mRNAs allow selective translation of key anti-apoptotic and adaptive proteins. These proteins promote cellular processes that sustain cell survival, among them metabolic reprogramming, redox balance, and epithelial-to-mesenchymal transition, thus facilitating tumor progression and therapy resistance. IRES activity is dynamically regulated by IRES trans-acting factors, such as YB-1, PTB, and hnRNPA1, which respond to cellular stress by enhancing translation of crucial mRNAs. Emerging therapeutic strategies include pharmacological IRES inhibitors, RNA-based approaches targeting ITAF interactions, and IRES-containing vectors for controlled therapeutic gene expression. A deeper understanding of translational reprogramming, IRES structural diversity, and ITAF function is essential to develop targeted interventions to overcome therapeutic resistance and eliminate persistent tumor cell populations.

## Simple Summary

Tumors grow in hostile environments where oxygen and nutrients are limited, and chemotherapy adds further stress. Under these conditions, cancer cells suppress normal protein production but activate an alternative mechanism called IRES-mediated translation. IRES elements functionally allow ribosomes to continue producing essential proteins when normal translation is impaired. This cap-independent translation mechanism permits cancer cells to produce key survival proteins, such as BCL2, XIAP, and NRF2, which are essential to block cell death, resist chemotherapy, and adapt to stressful conditions. IRES-mediate translation supports metabolic reprogramming and other cellular processes such as cancer cell plasticity, epithelial-to-mesenchymal transition, and cell survival. Understanding the mechanism of IRES-mediated translation may open new perspectives for therapies against aggressive and therapy-resistant tumors.

## 1. Introduction

Translational control is a mechanism by which cells adapt protein synthesis to adverse environmental conditions. Among alternative translation pathways, internal ribosome entry sites (IRESs) allow the translation of a class of mRNAs when cap-dependent translation is suppressed. Despite growing evidence of the involvement of IRESs in cancer biology, their precise role in tumor adaptation to the microenvironment and their potential as therapeutic targets remain areas of active research. This review provides a comprehensive overview of IRES-mediated translation (IMT) as an adaptive response to stresses present in the tumor microenvironment (TME) such as hypoxia and nutrient deprivation, and discusses their implications for tumor plasticity, therapy resistance, and novel therapeutic strategies.

## 2. IRES-Mediated Translation: Mechanisms and Regulation

### 2.1. Canonical vs. Non-Canonical Translation

In eukaryotic cells the initiation of canonical translation is mediated by the interaction of translation initiation factors with the cap structure, methylguanosine (m^7^G), present at the 5′ end of mRNA. According to this mechanism, the cap located at the 5′ end of mRNAs is recognized by eukaryotic initiation factor E (eIF4E), a component of the eIF4F complex [1]. Then, the eIF4F complex facilitates recruitment of the 40S ribosomal subunit and initiates the mRNA scanning toward the start codon, a process tightly regulated by nutrient and energy signals through the mTOR signaling pathway [2].

Hypoxia, oxidative stress, nutrient deprivation, endoplasmic reticulum (ER) stress, or DNA damage are stressful conditions that cause blockage of the cap-dependent translation, through eIF2α phosphorylation, mTOR inhibition, or disruption of eIF4E function [3,4]. In response, cells activate non-canonical translation pathways that enable selective protein synthesis even when global translation is suppressed. Among alternative pathways, IMT is one of the most extensively studied mechanisms [3,4]. IRESs are structured RNA elements located in the 5′ untranslated region (5′ UTR) of specific mRNAs, allowing direct ribosome recruitment independently of the 5′ cap structure [4,5]. Initially discovered in positive-strand RNA viruses [6], IMT has been identified in cellular mRNAs involved in stress response, apoptosis, cell cycle control, and tumor progression [7]. Other non-canonical mechanisms include upstream open reading frames (uORFs), leaky scanning, reinitiation, and m^6^A-mediated initiation. These translation mechanisms may coexist or compete within a single transcript, contributing to a complex landscape of translational regulation [7,8,9].

### 2.2. IRES Mechanisms and Regulation

IMT enables protein synthesis initiation without the need for cap recognition or ribosomal scanning [7,8,9]. IRES elements function through their secondary RNA structures, whose recognition is assisted by IRES trans-acting factors (ITAFs), a group of RNA-binding proteins that can stabilize or remodel the IRES to facilitate ribosome recruitment or interaction with the initiation machinery, such as eukaryotic initiation factor 3 (eIF3) or eIF4G [10]. The stressful conditions of the TME, such as hypoxia, oxidative stress, and limited nutrients, represent stimuli that activate IMT, ensuring continued expression of key survival and stress-adaptation proteins such as VEGF, c-Myc, BCL-2, and XIAP [7,8,9].

The function of many cellular IRESs is tightly regulated by specific ITAFs, which are influenced by stress signals, subcellular localization, and post-translational modifications. Among the ITAFs, some have been shown to play a significant role in stress response, cancer progression, and therapy resistance, including PTB (Polypyrimidine Tract Binding protein), hnRNP A1 and C1/C2, La autoantigen, and Y-box binding protein 1 (YB-1) [11,12,13].

**Figure 1:**
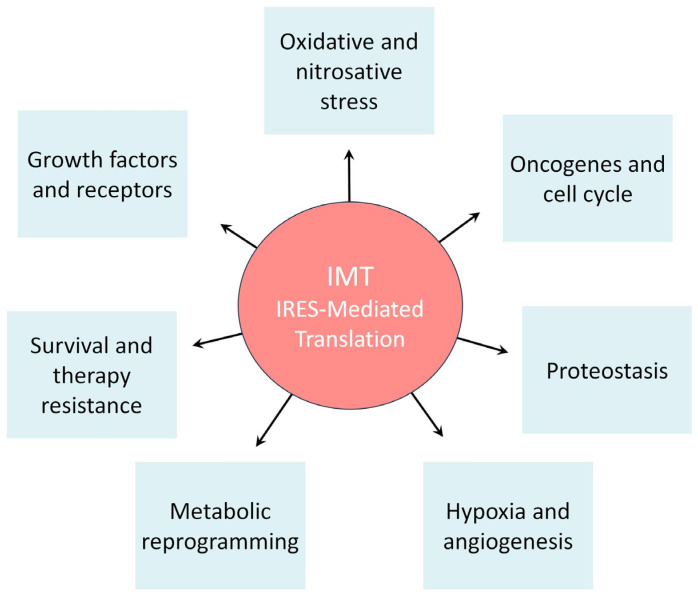
The central role of IRES-Mediated Translation (IMT) in orchestrating cancer cell responses to tumor microenvironment (TME) stress conditions. This figure illustrates how IMT allows cancer cells to adapt protein synthesis to stress conditions typical of the TME, such as hypoxia and nutrient deprivation. The surrounding boxes depict the major biological processes regulated by IMT that contribute to tumor proliferation and aggressiveness. Specifically, IMT controls the expression of key enzymes in one-carbon and polyamine metabolism, ensuring the continuous synthesis of nucleotides and the protection of DNA. It also regulates the expression of growth factors and oncogenes that promote cell cycle progression and survival. Furthermore, IMT modulates cellular stress response pathways, including the management of oxidative and nitrosative stress. Finally, IMT is a key factor in conferring therapy resistance, allowing tumor cells to maintain the expression of essential proteins even during treatment.

IMT promotes the synthesis of growth factors and oncogenes that drive cell cycle progression. It also orchestrates a complex metabolic reprogramming and regulates key pathways of one-carbon and polyamine metabolism, which are fundamental for supporting DNA synthesis and chromatin remodeling. Ultimately, IMT enables cancer cells to manage severe oxidative and nitrosative stress and to integrate into the broader cellular stress response, conferring a resilience leading to therapy resistance (Figure 1).

## 3. The Tumor Microenvironment (TME): Stress Conditions and Adaptive Translation

**Figure 2:**
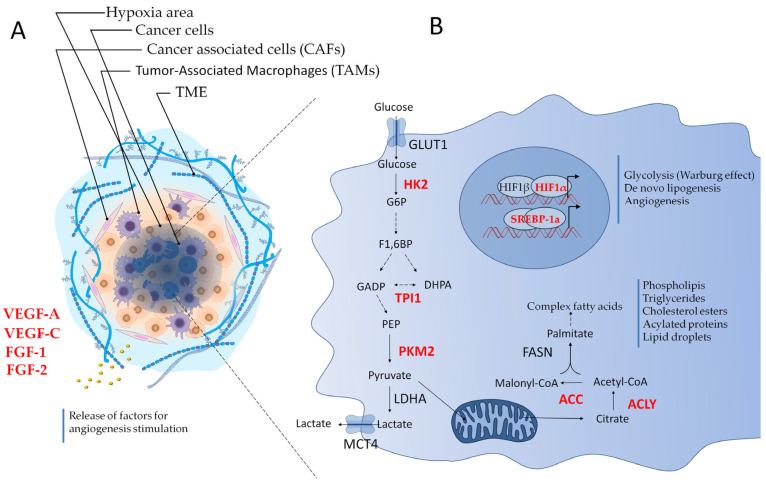
Interplay within the tumor microenvironment and IRES-dependent metabolic reprogramming in cancer cells. This figure illustrates (**A**) the key components of the tumor microenvironment (TME), including hypoxic areas, cancer cells, cancer-associated fibroblasts (CAFs), and tumor-associated macrophages (TAMs), highlighting their collective secretion of pro-angiogenic and pro-lymphangiogenic factors (VEGF-A, VEGF-C, FGF-1, FGF-2). (**B**) Detailed representation of glucose metabolism reprogramming in cancer cells, demonstrating increased glycolysis (Warburg effect) and de novo lipogenesis, crucial for tumor growth and proliferation. Notably, key enzymes and transcription factors indicated in red (e.g., HK2, TPI1, PKM2, ACLY, HIF1α, SREBP-1a) are frequently regulated through IRES-dependent translation mechanisms, allowing for their efficient expression under stress conditions prevalent in the TME, such as hypoxia. These proteins are essential for promoting the synthesis of phospholipids, triglycerides, cholesterol esters, acylated proteins, and lipid droplets, supporting the metabolic demands of cancer cells.

The intricate complexity of cancer becomes evident upon microscopic examination of solid tumors, revealing that the tumor microenvironment (TME) is a highly structured ecosystem containing a variety of non-malignant cell types alongside tumor cells, immersed in an altered and vascularized extracellular matrix (Figure 2A) [14,15]. The TME includes a rich diversity of immune cells, cancer-associated fibroblasts (CAFs), endothelial cells (ECs), pericytes, and other tissue-specific cell types, such as adipocytes and neurons [14,15]. Several studies conducted in vitro and preclinical tumor models show that TME cells and the molecules they secrete are considered key players in cancer pathogenesis and therefore represent attractive therapeutic targets [14,15].

Tumor cells orchestrate a favorable environment by recruiting and reprogramming non-tumor host cells and remodeling the vasculature and extracellular matrix (ECM).

There are multiple mechanisms through which this intercellular cross-talk is regulated, including cell–cell contact and paracrine signaling [14]. Contact-dependent communication is mediated by adhesion molecules, including integrins, cadherins, selectins, and members of the immunoglobulin superfamily, and via gap junctions. An example of contact-dependent intercellular signaling in the TME is the PD-L1/PD-1 pathway. Tumor cells frequently overexpress the immune checkpoint protein PD-L1, which interacts with the PD-1 receptor on adaptive immune cells to suppress immune surveillance. In addition to direct cell–cell contact, paracrine signaling involves the release of cytokines, chemokines, growth factors, and proteases. These molecules, secreted in response to cancer-intrinsic characteristics and cellular stress, originate from multiple cell types in the TME. They exert direct and indirect actions on target cells through binding to their receptors or via ECM remodeling [14].

Endothelial cells within the TME give rise to an aberrant, hypoperfused vasculature, often unable to meet the metabolic demands of rapidly proliferating tumors (Figure 2A) [16,17,18,19]. Hypoxia and nutrient deprivation act as potent stressors that directly impact mRNA translation [20,21]. For example, hypoxia promotes IMT by activating Hypoxia-Inducible Factor 1 alpha (HIF-1α) expression [21], through the cap-dependent translation suppression via 4E-BP1 dephosphorylation and eIF2α phosphorylation [22]. Nutrient deprivation, particularly of glucose and glutamine, inhibits mTORC1, further suppressing cap-dependent translation [23,24]. Oxidative stress and chronic inflammation elevate reactive oxygen species (ROS) levels, leading to DNA damage and activation of the integrated stress response (ISR), which involves PERK–eIF2α signaling [25]. Collectively, these stressors lead to global translational reprogramming and the selective translation of mRNAs containing IRES elements or structured 5′ UTRs, allowing continued production of proteins involved in angiogenesis (e.g., VEGF), metabolic adaptation (e.g., HIF-1α), and survival (e.g., BCL-2, XIAP). Cancer stem cells (CSCs) residing in hypoxic or nutrient-depleted niches could utilize IMT to maintain quiescence or resist therapy [26]. Similarly, EMT involves translational reprogramming that supports invasion and dissemination [27].

Recent research has underscored the critical role of mechanical forces and ECM remodeling in shaping tumor behavior. Changes in tissue stiffness can trigger signaling pathways, such as YAP/TAZ pathway, which drive tumor cell migration, invasion, and metastasis [28]. These mechanical interactions within the TME are now recognized as key contributors to tumor aggressiveness and therapeutic resistance. Moreover, extracellular matrix (ECM) proteins, such as thrombospondin-1 (TSP-1), and the balance between proteases and their inhibitors have also been implicated in tumor progression, metastasis, and response to therapy [29]. An additional layer of complexity is introduced by the functional plasticity of immune cells within the TME. Tumors can reprogram immune cells, such as TAMs, to support growth and evade immune surveillance [30]. The discovery of the adaptive potential of cancer cells highlights the dynamic nature of tumors and underscores the urgent need for therapies that can intercept or reverse this plasticity to improve patient outcomes [31].

## 4. IMT of Growth Factors and Their Receptors in the TME

During stressful conditions within the TME, tumor cells utilize IMT or other alternative mechanisms to selectively synthesize key growth factors and their receptors, which are crucial for sustaining mitogenic signaling, survival, and resistance to apoptosis under adverse conditions.

Among these, the insulin-like growth factor 1 receptor (IGF1R) functions as a central mediator of mitogenic and anti-apoptotic signaling [32]. IGF1R mRNA contains an extraordinarily long (1040 nucleotides) 5′ UTR that adopts a highly base-paired internal structure (dG > −500 kCal/mol), representing a substantial impediment to ribosome scanning. The 5′ UTR of human IGF1R enables an alternative mechanism for IGF1R translation initiation, allowing the 40S ribosome to circumvent the obstacles presented by the highly structured 5′ UTR. The central functional IRES is delimited by the 3′-terminal 90 nucleotides of the 5′ UTR, positioned immediately upstream of the start codon. Two ITAFs, HuR and hnRNP C, compete for interaction with the IRES and differentially regulate its activity. Pathological dysregulation of the IGF1R IRES has also been demonstrated in human breast cancer cells compared to non-transformed breast epithelial cells through changes in the activities of IRES regulatory RNA-binding proteins [33,34].

Insulin-like growth factor 2 (IGF2) is important for fetal growth and development. Human and murine IGF2 are encoded by a set of mRNAs that differ only in their 5′ UTR. In rapidly growing human rhabdomyosarcoma (RD) cells, the IGF2 mRNA designated leader 4 (L4) is constitutively translated, whereas the IGF2 mRNA designated leader 3 (L3) is translated in a rapamycin-inhibitable manner, through an IRES-mediated mechanism. The IGF2 mRNA-binding proteins (IMPs) have been retrieved by their ability to bind differentially to the 1.2-kb L3 but not the 0.1-kb L4 5′ UTR [35,36,37]. Moreover, it has been reported that mTORC1 regulates the translational initiation of the IGF2 L3 mRNA through a Raptor-independent phosphorylation of the RNA-binding protein IMP2. This phosphorylation enhances the ability of IMP2 to bind to the L3 5′ UTR, thereby enabling translation to be initiated via a cap- and eIF-4E-independent IMT mechanism [37].

In contrast, IMT of IGF1 has been less extensively characterized. However, emerging evidence from physiological contexts, such as skeletal muscle [38] and liver regeneration [39], suggests that IGF1 could also undergo cap-independent translation, a mechanism that may similarly be co-opted in tumors.

Another gene that harbors an IRES element is Gastrin, which encodes for a gastrointestinal peptide hormone [40]. Gastrin is normally expressed in G cells of the stomach antrum and regulates both acid secretion and proliferation of gastric mucosal cells. However, gastrin upregulation has been shown at both gene and protein levels in a number of gastrointestinal (GI) and non-GI cancers. Sustained gastrin production supports pro-survival and proliferative signaling through the CCK-2R receptor [41], contributing to tumor progression and chemoresistance, especially in gastric and colorectal cancers. As well as acting as a growth hormone, it has well-documented pro-angiogenic and anti-apoptotic properties [40,42]. A gastrin transcript expressed in a panel of GI cancer cell lines contains an IRES that has basal activity in both pancreatic and colon cancer cells. The 5′ UTR of this alternative gastrin transcript is GC-rich, in keeping with other described IRESs, which are generally highly structured [42].

The proto-oncogene SRC is a non-receptor tyrosine kinase involved in cell proliferation, invasion, metastasis, and angiogenesis [43,44]. The level of c-Src protein is known to increase in a variety of tumors. It activates STAT3, which transcriptionally regulates expression of Bcl-XL, c-Myc, and cyclin D1 leading to activation of anti-apoptotic and cell cycle progression pathways [45]. It has been shown that the activated c-Src-focal adhesion kinase complex promotes cell mobility, cell cycle progression, and cell survival. The c-Src activities are also important for promoting vascular endothelial growth factor (VEGF)-associated tumor angiogenesis and protease-associated metastasis [45]. Transcription of this gene from two different promoters and alternative splicing results in mature transcripts that differ only in the extreme 5′ ends but encode the same 60-kDa c-Src protein. Genetic analyses have permitted to define the IRES region between nt 200–383 of the c-Src mRNA, which harbors initiator AUG (at nt 351) [45].

## 5. IMT of Oncogenes Driving Cell Proliferation and Cycle Progression

Beyond growth factors and their receptors, IMT also governs the expression of key intracellular regulators that directly control cell proliferation and cell cycle progression.

Among the most studied are the MYC proto-oncogenes, c-Myc, L-Myc, and N-Myc, all containing functional IRESs in their 5′ UTRs [46]. The c-myc proto-oncogene regulates cellular metabolism, cell proliferation, and programmed cell death (apoptosis). Its expression is dysregulated in several cancers, with alterations affecting mRNA and protein stability mechanisms and the control of c-Myc translation. Different transcription start sites within the gene give rise to four transcripts, with the predominant mRNA having a structured 5′ UTR of ~400 nt containing an IRES element. Therefore, most c-myc mRNAs have the potential to initiate translation through IRES [47]. Although the IRES stimulates reporter gene protein synthesis in several cell lines tested, a 20-fold disparity was observed between HeLa and MCF7 cells, lines in which the IRES is more and less active, respectively. This cell-type-specific variation in IRES activity implies that the function of this element could be modulated by non-canonical transactivating factors. This is justified by the fact that the composition of the protein complexes involved in translation initiation depends on the tumor cell type considered [47]. N-Myc is a proto-oncogene whose expression increases through gene amplification in many neuroblastomas [48]. This gene was the first example of a mechanism for regulating gene expression at the level of protein synthesis. Gene reporter assays have identified the presence of an IRES in the 5′ UTR of N-myc [48]. This IRES is similar in length to that of the c-Myc gene and supports protein synthesis in cells of various neuroblastoma cell lines. Furthermore, the N-Myc IRES also drives translation in non-neuronal cells, with activity comparable to that observed in neuronal cells. The N-myc IRES is up to sevenfold more active than that of c-Myc, suggesting that neuronal-specific non-canonical transduction factors are utilized by N-myc but not by the c-myc IRES [48]. L-Myc mRNA also contains an IRES in its 5′ UTR. Like many other cellular IRESs, the L-myc IRES is highly structured and modular in nature, and the entire 5′ UTR is required for maximum IRES efficiency [48]. Unlike other structured IRESs, the ribosome entry window within the L-myc IRES is located some distance upstream of the start codon, and therefore, this IRES uses a “land and scan” mechanism to initiate translation [49]. Moreover, while translation of the c-myc and N-myc IRES mRNAs can involve both cap-dependent and internal initiation, cap-independent translation occurs for all translation initiations of the L-myc mRNA. Conserved RNA structural motifs in MYC family members indicate a shared regulatory mechanism; however, differences in activity exist that result from differential availability of ITAF in various cell types [50].

Lymphoid enhancer factor-1 (LEF-1), a LEF/TCF transcription factor, mediates WNT signaling control of the cell cycle and differentiation by recruiting the transcription activator β-catenin to target genes. LEF1 is aberrantly expressed in >80% of tumors with mutated WNT signaling components and causes hyperactive, constitutive signaling and cellular transformation [51]. The mRNA with the long 5′ UTR is aberrantly transcribed, whereas the shorter promoter producing LEF1 mRNA is not expressed. Loss of balanced expression has significant effects on cancer progression [51,52]. Transfection experiments performed with a dicistronic vector have demonstrated that the long 5′ UTR contains an IRES. Furthermore, 5′ UTR deletion analysis shows that maximal IRES activity requires most of the 5′ UTR, consistent with the idea that cellular IRESs require multiple modules for efficient activity [51]. In particular, eIF4A, an RNA helicase, induces potent non-canonical effects on the LEF1 IRES, and inhibition of eIF4A by hippuristanol blocks IRES mRNA translation and triggers dissociation from polyribosomes [52].

Members of the HOX gene family, essential for developmental programming, also exhibit IRES activity in their 5′ UTRs. For example, HOXA3 can be selectively translated under conditions of growth arrest or stress [53,54]. This selective translation appears to be regulated by ribosomal proteins such as RPL38 [55], which modulate the expression of specific HOX proteins and may contribute to tumor progression through aberrant signaling during development.

Another key example is CCND1, which encodes cyclin D1 involved in cell cycle progression by inducing the G1-S transition through the activation of cyclin-dependent kinases, Cdk4 and Cdk6. Cyclin D1 overexpression is frequently found in tumors, including breast cancer, and the frequency of CCND1 amplification is between 9 and 15%. CCND1 amplification is associated with an increased risk of tumor recurrence and reduced chemosensitivity. In breast cancer, there is also an association between CCND1 amplification and elevated proliferation, high histopathological grade, and the Luminal B subtype [56,57,58,59]. This translational control likely contributes to tumor cell survival and uncontrolled proliferation [56,57,58,59]. The CCND1 gene contains an IRES that is regulated by AKT activity, potentiated by rapamycin through a p38 MAPK and ERK-dependent pathway, and transactivated by the autoantigen La ITAF [56,57,58,59].

## 6. IMT Within the Integrated Stress Response (ISR) and Proteostasis Regulation in the TME

Tumor cells adapt to hostile microenvironmental conditions through translational control, with the Integrated Stress Response (ISR) playing a central role in modulating protein synthesis and preserving proteostasis [25,60,61]. A hallmark of the ISR is the phosphorylation of eukaryotic initiation factor 2 alpha (eIF2α) by stress-activated kinases, PERK, GCN2, PKR, and HRI, leading to a global inhibition of cap-dependent translation and selective protein synthesis via IRES-mediated initiation [3,62,63].

One key effector of proteostasis in ISR context is BiP/GRP78 (Binding Immunoglobulin Protein/Glucose-Regulated Protein 78), a chaperone that maintains protein folding accuracy within the ER. In cancer cells, high protein synthesis rates often cause ER stress due to the accumulation of misfolded proteins, activating the unfolded protein response (UPR) through BiP dissociation from ER sensors PERK, IRE1, and ATF6 [25,64]. PERK activation triggers eIF2α phosphorylation, represses global translation, and enhances cap-independent translation of stress-responsive mRNAs, including ATF4, CHOP, and BiP itself, via IRES elements in their 5′ UTRs [60,65].

BiP/GRP78 is a key initiator of the UPR and acts as a major regulator of protein homeostasis. Under ER stress, its expression is upregulated at both the transcriptional and translational levels, and it contributes to tumor cell survival by facilitating correct protein folding and promoting ER-associated degradation (ERAD) [25]. Specifically, BiP binds to exposed hydrophobic domains of non-native proteins, preventing aggregation and enabling proper folding [25]. Moreover, BiP/GRP78 suppresses apoptosis by modulating the PERK–eIF2α–CHOP axis and interacting with apoptotic regulators, such as procaspase-7 and BCL2 family members, further supporting tumor survival under proteotoxic stress [25,66,67,68]. The presence of BiP at the cell surface (surface BiP, sBiP) contributes to oncogenic processes, including proliferation, immune evasion, and therapy resistance, possibly via interactions with co-receptors such as Cripto [69,70].

Heat shock proteins (HSPs), such as HSP70, also use IMT to maintain protein homeostasis under stress conditions in the TME. Their cap-independent synthesis ensures adequate chaperone levels necessary for tumor cell survival [71].

## 7. IMT in Hypoxia and Tumor Angiogenesis

Within the TME, hypoxia emerges as a consequence of disorganized and inefficient neovasculature, unable to meet the oxygen demands of proliferating cancer cells. This deficiency activates a transcriptional program centered on HIF-1α, which governs key processes such as angiogenesis, glycolysis, and survival (Figure 2A) [16,72].

The mRNA of HIF-1α contains a functional IRES [73] that enables its translation under hypoxic conditions. This IRES contains complex secondary structures that recruit ribosomes through interactions with RNA-binding proteins. One such ITAF is polypyrimidine tract-binding protein (PTB), which enhances HIF-1α translation during hypoxia by binding its 5′ UTR [74]. In hypoxic conditions, HIF1A translation proceeds through a 4EBP1/eIF4G-dependent, cap-independent pathway [74,75]. The RNA-binding protein YB-1 further supports this process by stabilizing HIF-1α IRES structure and facilitating ribosome recruitment [76]. Elevated YB-1 levels during hypoxia correlate with sustained HIF-1α expression and aggressive tumor phenotypes, suggesting YB-1 as a potential therapeutic target [77].

Among other hypoxia-responsive mRNAs, VEGF-A is one of the most well-characterized in terms of IMT (Figure 2A). Its 5′ UTR contains two independent IRESs, IRES A and IRES B. IRES A initiates at the canonical AUG, producing classical VEGF-A isoforms, while IRES B initiates at an upstream CUG codon, generating a longer L-VEGF-A isoform with distinct functions [78,79,80,81]. While VEGF-C translation is also known to be IRES-dependent, the role of IRESs in VEGF-D translation remains less well defined and warrants further investigation [82,83].

Hypoxia also influences the translation of Delta-Like 4 (DLL4), which helps shape vascular architecture in tumors by modulating the balance between endothelial tip and stalk cells through Notch signaling [84]. Interestingly, DLL4 5′ UTR harbors an IRES which is strongly activated under ER stress via the PERK/eIF2α arm of the UPR [85].

Again, both FGF-1 and FGF-2 (fibroblast growth factor 1 and 2) (Figure 2A) also possess a functional IRES that allows translation under hypoxia and contributes to feedback activation of HIF-1α (Figure 2B) [86,87].

## 8. Metabolic Adaptations for Tumor Survival: The Role of IMT

Tumor cell metabolism undergoes profound reprogramming to adapt to hypoxia, nutrient deprivation, and stress within the TME [88,89]. This adaptation involves metabolic pathways such as anaerobic glycolysis (Warburg effect) [90], glutaminolysis [91], lipogenesis [92], and one-carbon metabolism [93].

Many proteins involved in these pathways are translated via IRESs, enabling tumor cells to sustain protein synthesis under conditions that impair canonical cap-dependent translation.

### 8.1. Glycolysis and the Warburg Effect

In cancer cells, glycolysis supports continuous ATP production and biosynthetic activity, even in the presence of oxygen, through the Warburg effect. This metabolic shift allows cancer cells to proliferate rapidly and adapt to the hostile microenvironment. The proto-oncogene c-Myc orchestrates a broad transcriptional program that enhances the expression of glucose transporters (GLUT1/SLC2A1), hexokinase 2 (HK2), phosphofructokinase isoforms (PFKM, PFKP, and possibly PFKL), enolase 1 (ENO1), and lactate dehydrogenase A (LDHA), thereby sustaining glycolytic flux (Figure 2B) [90]. It also upregulates monocarboxylate transporters MCT1 and MCT4 to facilitate lactate export and preserve redox balance [94].

At the translational level, several glycolytic genes are regulated via IRES elements in their 5′ UTRs, allowing continued protein synthesis under stress (Figure 2B). One such example is the PKM2 gene, which encodes a pyruvate kinase isoform central to tumor metabolism. Its mRNA undergoes alternative splicing promoted by hnRNPA1, hnRNPA2, and PTB1, resulting in the inclusion of an IRES element that enables translation during adverse conditions [95]. PKM2 catalyzes the final step of glycolysis, converting phosphoenolpyruvate (PEP) to pyruvate and generating ATP. In cancer cells, PKM2 predominantly exists in a dimeric form with low enzymatic activity, which slows down glycolytic flux and allows accumulation of upstream intermediates that are diverted into anabolic pathways. Additionally, dimeric PKM2 can translocate to the nucleus and function as a transcriptional coactivator, promoting tumor progression under stress conditions.

Recent work by Ismail et al. (2025) identified functional IRES elements in the 5′ UTR of HK2 mRNA, which drive its cap-independent translation in glioma cells under hypoxia [96]. HK2 encodes hexokinase 2, the enzyme that catalyzes the first step of glycolysis by phosphorylating glucose to glucose-6-phosphate. Its overexpression in tumors facilitates high glycolytic flux and supports anabolic metabolism. Similarly, an IRES has been characterized in triosephosphate isomerase 1 (TPI1) gene [96], which encodes a glycolytic enzyme that interconverts dihydroxyacetone phosphate and glyceraldehyde 3-phosphate. Other glycolytic enzymes, such as LDHA, ENO1, and 6-phosphofructo-2-kinase/fructose-2,6-bisphosphatase 3 (PFKFB3), also maintain high protein levels under hypoxia or nutrient deprivation, despite global inhibition of translation. Although IRES activity has not been directly demonstrated in their mRNAs, persistent protein expression, association with polysomes, and discrepancies between mRNA and protein levels suggest that alternative translational mechanisms may be involved [97]. LDHA, for example, catalyzes pyruvate-to-lactate conversion to regenerate NAD^+^, a process critical for anaerobic glycolysis and frequently upregulated in cancer [98]. ENO1, beyond its glycolytic function, has been implicated in cancer cell migration, immune evasion, and chemoresistance, and remains highly expressed under stress [99,100]. Analogously, PFKFB3, a potent glycolysis regulator and target of HIF-1α, is also overexpressed in hypoxic tumors [101]. Given the critical role of these genes in sustaining glycolysis and the Warburg effect, along with their consistently high expression in cancer cells, it is plausible that their upregulation may also occur at the translational level through IRES-mediated mechanisms. However, this possibility warrants further investigation to elucidate the contribution of IMT in tumor metabolism.

### 8.2. De Novo Lipogenesis

Increased de novo lipid synthesis is another key metabolic adaptation in cancer, enabling membrane biogenesis, energy storage, and redox homeostasis [102]. This process is regulated by sterol regulatory element-binding protein 1 (SREBP-1), the master transcription factor involved in the regulation of lipogenic gene expression. The SREBF1 gene gives rise to SREBP-1a and SREBP-1c, which arise through the use of alternative promoters that produce transcripts in which distinct first exons are spliced into a common second exon. SREBP-1 is synthesized as inactive precursors anchored to ER membranes. After proteolytic cleavage in the Golgi apparatus, the N-terminal active transcription factor translocates into the nuclei to transactivate lipogenic and cholesterologenic genes, including acetyl-CoA carboxylase, fatty acid synthase, and stearoyl-CoA desaturase. Importantly, SREBP-1 participates in the metabolic reprogramming of various tumors and has been a biomarker for prognosis or drug efficacy for cancer patients. Luciferase assay with dicistronic vectors localized an IRES in the 5′ UTR of SREBP-1a, which is activated under conditions of serum deprivation and ER stress (Figure 2B) [92]. Another study showed that IMT of SREBP-1a mRNA promotes lipid droplet (LD) accumulation [103,104], which in turn creates a stress condition triggering SREBP-1a IRES activity. Accumulation of lipid droplets supports tumor proliferation by storing and mobilizing neutral lipids and contributes to cellular homeostasis under stress. Several studies have shown that genetic silencing or pharmacological inhibition of SREBP-1 leads to apoptotic cell death in various tumor types, highlighting its potential as a therapeutic target [105,106].

Since many tumors exhibit a “lipogenic” phenotype, it was hypothesized that, in addition to the transcription factor SREBP-1, the expression of some lipogenic genes might be controlled at the translational level. Subsequent studies demonstrated that key lipogenic enzymes such as ATP-citrate lyase (ACLY) and acetyl-CoA carboxylase alpha (ACC1), central to this metabolic pathway, possess an IRES within their respective mRNAs [107,108,109,110]. ACLY catalyzes the conversion of cytosolic citrate into oxaloacetate and acetyl-CoA, which is a precursor for fatty acid synthesis. Moreover, acetyl-CoA is used in histone acetylation and thus ACLY plays a key link between metabolism and gene regulation at the epigenetic level [107]. Acetyl-CoA also contributes to the acetylation of proteins playing key roles in promoting tumor aggressiveness, metastasis chemoresistance [107,108,109].

ACC1, the rate-limiting enzyme in de novo fatty acid synthesis, catalyzes the carboxylation of acetyl-CoA to malonyl-CoA. ACC1 is considered a tumor marker as it is frequently overexpressed in various cancers and contributes to metabolic reprogramming and epigenetic remodeling [111,112,113]. Interestingly, stressful conditions in the TME induce significant ACC1 activity, creating conditions for increased fatty acid synthesis and their accumulation in lipid droplets. This accumulation inhibits fatty acid beta-oxidation, which is essential for the immunosurveillance activity of CD8^+^ T cells, thus weakening antitumor immune responses and facilitating immune evasion [114].

Transcription of the ACACA gene produces three distinct transcripts, owing to the use of distinct promoters and alternative splicing. Among these transcripts, the most abundant contains an IRES in the 5′ UTR, as confirmed by assays with dicistronic constructs. Treatment of hepatoma cells (HepG2) with tunicamycin and thapsigargin, ER stressors, results in an increase in IMT of ACC1 mRNA. Importantly, chemically induced hypoxia also triggers IMT of ACC1 mRNA, supporting the activation of lipogenesis under this stressful condition [115].

Other lipogenic genes, including SLC25A1, FASN, GPATs, and DGATs, are upregulated in tumors and contribute to metabolic reprogramming [102,116,117]. Although direct evidence for IRES activity in these genes remains limited, their persistent expression under translation-inhibitory conditions suggests the involvement of alternative mechanisms worth further investigation [111,112,113].

### 8.3. IMT Control of One-Carbon and Polyamine Metabolism in Cancer

**Figure 3:**
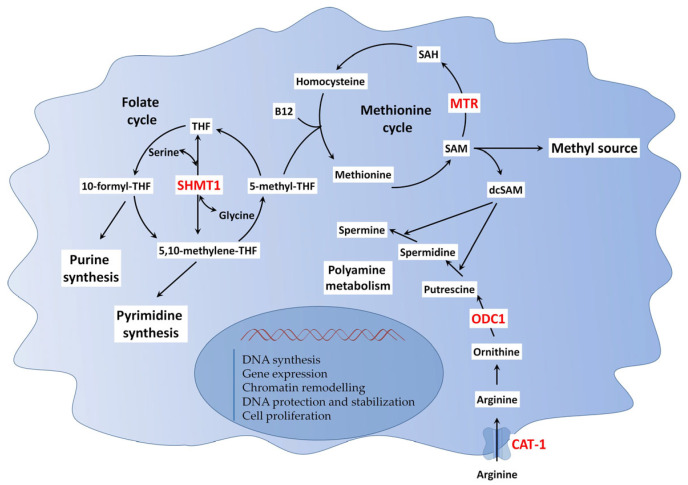
Role of IMT in the interplay between one-carbon and polyamine metabolisms, supporting DNA synthesis and rapid cancer cell proliferation. This figure provides a detailed representation of the interplay between one-carbon metabolism (including the folate and methionine cycles) and polyamine biosynthesis. These pathways are crucial for sustaining rapid cancer cell proliferation by enabling the enhanced synthesis of key biomolecules. They provide purine and pyrimidine nucleotides essential for DNA replication and gene transcription, and S-adenosylmethionine (SAM), the universal methyl donor required for epigenetic regulation, including chromatin remodeling. Additionally, they produce polyamines, which are fundamental for DNA stabilization and protection. Notably, key genes indicated in red (SHMT1, ODC1, MTR, and CAT-1) are regulated through IMT, a mechanism that allows for their efficient expression under the high-stress conditions prevalent in the TME.

The one-carbon (1C) metabolism and polyamine biosynthesis are two interconnected metabolic pathways that sustain tumor growth by supporting nucleotide synthesis, chromatin regulation, and cellular proliferation (Figure 3) [118,119]. A central enzyme in the cytosolic branch of 1C metabolism is Serine hydroxymethyltransferase 1 (SHMT1), which catalyzes the conversion of serine to glycine and generates 5,10-methylene-tetrahydrofolate (5,10-methylene-THF). This compound donates one-carbon units required for de novo thymidylate synthesis via thymidylate synthase (TYMS) and indirectly contributes to the regeneration of methionine from homocysteine through 5-methyl-THF. This reaction is catalyzed by methionine synthase (MTR), a key enzyme connecting the folate and methionine cycles.

Methionine is subsequently converted into S-adenosylmethionine (SAM), the universal methyl donor required for the methylation of DNA, RNA, and histone proteins, processes that are central to epigenetic regulation in cancer [120]. In addition to its role in methylation, SAM is also decarboxylated to produce decarboxylated SAM (dcSAM), which is the aminopropyl donor in the synthesis of spermidine and spermine from putrescine. Thus, SHMT1, by providing one-carbon units, links nucleotide production directly to metabolic pathways that support methylation capacity and polyamine biosynthesis in tumors. This network is centered on key enzymes like methionine synthase (MTR), which catalyzes the regeneration of methionine from homocysteine [121]. By ensuring a constant supply of methionine, MTR guarantees the production of S-adenosylmethionine (SAM), thereby indirectly supporting multiple biosynthetic demands of proliferating cancer cells (Figure 3).

Studies reported that SHMT1 expression is regulated at the translational level by an IRES within its mRNA, which facilitates IMT under conditions of cellular stress. RNA-binding proteins such as CUGBP1 and hnRNP H2 mediate this process by bridging the 5′ and 3′ UTRs, maintaining SHMT1 protein levels during genotoxic insults such as UV damage [122,123]. SHMT1 is frequently overexpressed in tumors including breast, hepatocellular, and lung carcinomas, and is associated with poor prognosis [124].

The human MTR 5′ UTR is 394 bases long with an extensive secondary structure that is thought to inhibit ribosome scanning, raising the possibility that IRES-dependent initiation could be important for this leader-burdened mRNA [125]. The minimal IRES element spans 71 nucleotides immediately upstream of the initiation codon. Electrophoretic mobility shift analysis revealed the presence of a B12-dependent protein-RNA complex, suggesting the B12-dependent increase in IRES efficiency is mediated via an uncharacterized ITAF [125].

Downstream of the one-carbon cycle, polyamine biosynthesis also relies on IRES-mediated translational control to sustain the expression of key enzymes under stress. Central to polyamine biosynthesis are cationic amino acid transporter 1 (CAT-1, encoded by SLC7A1) which facilitates cellular uptake of arginine and ornithine, substrates for polyamine synthesis, and ornithine decarboxylase 1 (ODC1), the rate-limiting enzyme catalyzing ornithine decarboxylation to putrescine. This is further converted to spermidine and spermine through the sequential addition of aminopropyl groups donated by dcSAM. These polyamines promote chromatin remodeling, stabilize and protect DNA, contributing to tumor aggressiveness and resistance to apoptosis [118].

Both ODC-1 and CAT-1 are frequently overexpressed in malignancies such as colorectal, breast, prostate, and lung cancers, correlating with tumor aggressiveness and poor prognosis [118,126,127,128,129]. Notably, ODC1 and CAT-1 mRNAs contain IRES elements in their 5′ UTRs, enabling cap-independent translation when canonical initiation is suppressed [130,131,132].

## 9. IRES-Driven Translation in Tumor Cell Survival, Senescence, and Therapy Resistance

**Figure 4:**
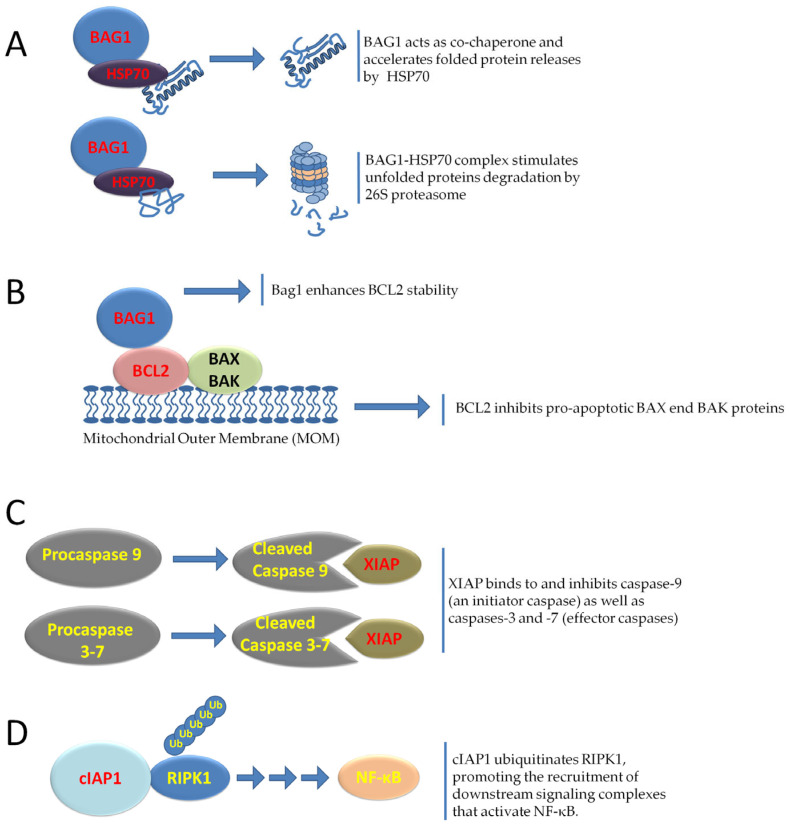
Diverse molecular mechanisms regulating cell survival and apoptosis. This figure illustrates several key molecular interactions contributing to cellular anti-apoptotic and pro-survival pathways. (**A**) BAG1’s Chaperone and Degradation Roles: BAG1 (Bcl-2-associated athanogene 1) interacts with HSP70 (heat shock protein 70). As a co-chaperone, BAG1 facilitates the release of properly folded proteins from HSP70. Additionally, the BAG1-HSP70 complex can target unfolded proteins for degradation by the 26S proteasome, preventing the accumulation of misfolded proteins. (**B**) BAG1’s Stabilization of BCL2: BAG1 enhances the stability of the anti-apoptotic protein BCL2, which is localized on the mitochondrial outer membrane (MOM). BCL2, in turn, inhibits the pro-apoptotic BAX and BAK proteins, thereby preventing mitochondrial outer membrane permeabilization and the intrinsic apoptotic pathway. (**C**) Caspase Inhibition by XIAP: X-linked inhibitor of apoptosis protein (XIAP) directly binds to and inhibits both initiator caspases (e.g., cleaved caspase-9) and effector caspases (e.g., cleaved caspases-3 and -7), thus blocking the execution phase of apoptosis. (**D**) NF-κB Activation by cIAP1: cellular inhibitor of apoptosis protein 1 (cIAP1) ubiquitinates RIPK1 (receptor-interacting protein kinase 1), which promotes the recruitment of downstream signaling complexes leading to the activation of NF-κB, a crucial transcription factor involved in cell survival, proliferation, and inflammation.

Due to abnormal vascularization, the lack of oxygen and nutrients can lead to tumor cell death. Despite the adverse conditions imposed by the tumor environment, tumor cells have developed a resilience program that ensures their survival through the expression of anti-apoptotic genes, including Bcl-2 Associated athanogene 1 (BAG1), BIRC2, XIAP, and members of the BCL2 family, thus enabling continued survival even under adverse conditions [133] (Figure 4).

BAG1 (Bcl-2-associated athanogene 1) is a multifunctional protein involved in the regulation of apoptosis, protein homeostasis, and cellular responses to stress. There are three main isoforms of BAG-1 in mammalian cells, designated BAG-1L (p50), BAG-1M (p46), and BAG-1S (p36), which act as pro-survival proteins and are associated with tumorigenesis and chemoresistance. The synthesis of BAG-1S depends on the presence of IRES in the 5′ UTR of the BAG-1 mRNA and requires the intervention of two ITAFs, poly (rC) binding protein 1 (PCBP1) and polypyrimidine tract-binding protein (PTB), for its function [134]. Studies have shown that BAG-1 IRES maintains synthesis of BAG-1 protein following exposure of cells to the chemotoxic drug vincristine but not to cisplatin and that this is brought about, in part, by the re-localization of PTB and PCBP1 from the nucleus to the cytoplasm [134].

The role of BAG1 in cell survival is further supported by its direct interactions with key anti-apoptotic and chaperone proteins. The BAG1 gene was originally discovered in a mouse embryo cDNA library screen using recombinant human BCL-2 protein as bait to identify novel BCL-2-interacting partners [135]. However, the main function of BAG1 is as a co-chaperone in conjunction with HSP70. HSP70 assists protein folding through ATP-driven cycles of substrate binding and release. HSP70 adopts an open conformation when ATP is bound to its nucleotide-binding domain, allowing for dynamic adaptation of the client protein within the substrate-binding domain. Conversely, ATP hydrolysis causes a structural conformational change that locks the hydrophobic region of the protein. Other factors, nucleotide exchange factors (NEFs), accelerate ADP dissociation and HSP70 opening, thus promoting client dissociation. In this context, BAG1 acts as a NEF, facilitating ADP release and ATP binding to HSP70 and modulating its activity in protein folding, substrate release and quality control [135]. This cooperation contributes to the cytoprotective role of HSP70, preventing protein aggregation and inhibiting apoptosome formation via interaction with APAF-1 [136].

XIAP (BIRC4) and cIAP1 (BIRC2) are key members of the Inhibitor of Apoptosis Protein (IAP) family, which act as cell survival and apoptosis regulators [135]. XIAP is the only IAP molecule showing a strong affinity for the direct binding and inhibition of caspases, through specific BIR1-3 (baculovirus IAP repeat) domains containing three cysteine residues and one histidine residue coordinating a Zn^2+^ ion. The activity of effector caspases, specifically caspase-3 and -7, is inhibited by binding the BIR1-BIR2 linker region of XIAP to their active sites, thus preventing the cleavage of the linker. The BIR3 domain of XIAP binds to the IAP-binding motif of caspase-9, located just above the active site, the exposure of which occurs during the proteolytic transformation of pro-caspase-9, resulting in restricted access to substrates. Moreover, the BIR1 domain is involved in the activation of the pro-survival NF-κB pathway [137,138].

Along with other IAPs, cIAP1 is an inhibitor of the extrinsic apoptosis pathway and supports tumor cell survival in a different manner than XIAP. cIAP1 possesses E3 ubiquitin ligase activity, through which it regulates RIP1 (Receptor-Interacting Protein Kinase 1) [139]. cIAP1-mediated ubiquitination of RIP1 creates a scaffold for the recruitment of the TAK1 (TGF-β activated kinase 1) complex [139]. TAK1 subsequently activates the IKK complex, leading to the nuclear translocation of NF-κB and the transcription of anti-apoptotic and inflammatory genes [140]. The overexpression of cIAPs observed in tumors reinforces the pro-survival NF-κB pathway, making the cIAP-RIP1-TAK1-NF-κB axis a target for therapeutic intervention aimed at re-sensitizing cancer cells to death. Its overexpression is linked to tumorigenesis and apoptosis inhibition in cancers such as gallbladder, ovarian, and hepatocellular carcinoma [139].

Translation of these anti-apoptotic proteins can be regulated by IRESs within their 5′ UTR. One study reported the identification of a 150 bp long IRES located proximal to the initiation codon (AUG) of the major open reading frame (ORF). This IRES enhances translation of cIAP1 mRNA following ER stress. An upstream ORF (uORF) whose initiation codon CUG bisects the IRES encodes a nonfunctional 21-amino acid peptide and acts to repress translation of cIAP1 mRNA under normal physiological conditions [141]. The activity of the cIAP1 IRES is regulated by specific ITAFs, including p86, a protein formed by the cleavage of p97/DAP5 during ER stress. Using RNA affinity chromatography, additional IRES-binding proteins of cIAP1 have been identified: NF45, NF90, IGF2BP1, and RHA. NF45 has also been shown to be required for cIAP1 induction during the ER stress response [141].

The existence of an auxiliary translation initiation complex has been suggested to be active during periods when the cell has reduced overall protein synthesis, which allows for continued translation of this important regulator of apoptosis and NF-κB signaling [141].

XIAP translation is mediated by a 162-nucleotide IRES element located in the 1.7 kb long 5′ UTR region, thus allowing de novo synthesis of XIAP during cellular stress and apoptosis [142]. Thus, by exploiting an IRES-dependent translation mechanism, new XIAPs can be produced when it is necessary to block or delay the progression of apoptosis [142]. Three ITAF/RNPs, namely La, hnRNP C1/C2 and hnRNP A1, have been identified to regulate the IRES activity of XIAP. IMT of XIAP mRNA contributes to cell survival under stress and cancer progression by ensuring continuous production of the protein, thus promoting resistance to apoptosis [142].

BCL2 (B-cell lymphoma type 2) and BCL-XL (extra-large B-cell lymphoma) are anti-apoptotic proteins that inhibit mitochondrial outer membrane permeabilization (MOMP) in the intrinsic apoptotic pathway. They act by suppressing pro-apoptotic effectors such as BAX and BAK, which form pores in the mitochondrial membrane, allowing the release of cytochrome c and activation of the caspase cascade. BCL2 and BCL-XL also regulate other members of the BCL2 family such as MCL-1 and A1, maintaining a balance between pro- and anti-apoptotic signals to ensure cell survival [143]. Both BCL2 and BCL-XL are overexpressed in several cancers and contribute to tumor cell survival and therapy resistance by preventing apoptosis in response to DNA damage, oxidative stress, or cytotoxic treatments. Translation of BCL2 and BCL-XL mRNAs is regulated, particularly through IRES elements located in their 5′ UTRs [144,145]. The Bcl2 IRES was discovered using bicistronic reporter assays and was confirmed by transfecting Bcl2 mRNA directly into cells. The authors went on to show that apoptosis induced by chemotherapeutic agents caused a 3–6-fold induction of IRES activity. IRES-mediated translation may represent a means by which apoptosis can be delayed or by which the cell can be rescued if the apoptotic stress is removed [144]. Of the other BCL2 family pro-survival proteins, Bcl-XL has also been proved to contain an IRES [145], but Bcl2A1, Mcl1 and Bcl-w are unlikely to possess this element because they all have short 5 UTRs. BCL2 and BCL-XL have been reported to be frequently overexpressed in senescent tumor cells to prevent their apoptotic clearance. Senescence is a stable and generally irreversible growth arrest triggered by various stressors, including oncogene activation, DNA damage, and therapy-induced injury [146,147]. Although senescent cells cease to proliferate, they remain metabolically active and secrete a broad range of cytokines, chemokines, growth factors, and matrix-remodeling enzymes, collectively referred to as the senescence-associated secretory phenotype (SASP), which profoundly remodels the tumor microenvironment [146,147]. While senescence initially serves as a tumor suppressor mechanism by arresting the proliferation of damaged cells [148], the chronic accumulation of senescent cells and their SASP factors can paradoxically promote tumor progression, inflammation, and resistance to therapy [149,150,151,152].

## 10. The Role of IMT in NRF2 and NOS2 Expression in Response to Oxidative and Nitrosative Stress in Cancer

Oxidative stress and redox imbalance are hallmarks of the TME, resulting from rapid proliferation, hypoxia, and metabolic dysregulation that contribute to increased ROS levels [153]. To prevent excess ROS from causing damage to DNA, proteins, and lipids, tumor cells activate adaptive mechanisms to maintain the synthesis of proteins essential for antioxidant defense, DNA repair, and overall survival. Among the key regulators of these responses, NRF2 (Nuclear Factor Erythroid 2-Related Factor 2) plays a key role in orchestrating antioxidant defense by activating the expression of genes containing antioxidant response elements (AREs) in their promoter. NRF2-responsive genes include heme oxygenase-1 (HO-1), NAD(P)H quinone oxidoreductase 1 (NQO1), and the catalytic subunit of glutamate cysteine ligase (GCLC). While NRF2 controls the antioxidant response, oxidants are a major inducer of NRF2 protein and activity. The human NRF2 gene encodes a 555-nucleotide mRNA strand of 5′ UTR containing 70% G and C. A stable secondary structure containing “stem and loops” was predicted, suggesting that NRF2 5′ UTR contains an IRES, enabling translation of NRF2 protein under oxidative stress [154]. Studies have reported that NRF2 mRNA contains an IRES within its 5′ UTR, enabling IMT during oxidative stress, and allowing the rapid accumulation and nuclear translocation of NRF2 to restore redox homeostasis [154]. Using tandem liquid chromatography MS, it has been highlighted that La autoantigen protein is an ITAF capable of binding and transactivating NRF2 5′ UTR in response to oxidative stress. In vitro RNA binding and in vivo ribonucleoprotein immunoprecipitation experiments showed that H_2_O_2_ dose- and time-dependent increases in La autoantigen binding to NRF2 5′ UTR [155]. Paradoxically, this activity also contributes to resistance to chemotherapy and radiotherapy, allowing tumor cells to survive therapeutic stress [156].

Inducible nitric oxide synthase (iNOS), encoded by the NOS2 gene, is an enzyme responsible for the production of nitric oxide (NO) in response to inflammatory stimuli. In the TME, iNOS expression is dysregulated, with its activity contributing to cancer progression [157,158]. At low to moderate concentrations, NO stimulates tumor cell growth, increases tumor invasion and metastatic potential, and accelerates angiogenesis by modulating vascular remodeling and the expression of pro-angiogenic factors such as VEGF [157,158,159,160,161]. Furthermore, NO influences signaling pathways related to cell survival and stress responses, further supporting tumor progression. In addition to its direct effects on tumor cells and the vasculature, iNOS-derived NO also plays a role in immune suppression within the TME [157,158,159,160,161]. This immunosuppressive action makes iNOS a key target for restoring immune surveillance in cancer. However, at high concentrations of NO, cytotoxic and cytostatic effects are observed, leading to macromolecular damage and triggering apoptosis or other forms of tumor cell death [157]. The dual nature of NO presents both challenges and opportunities for therapeutic intervention. Similarly to NRF2, NOS2 mRNA translation is regulated by an IRES [162]. Within the inflammatory and hypoxic TME, IMT of NOS2 allows for sustained NO production, even under conditions where cap-dependent translation is suppressed. This persistent NO generation contributes to altered redox states, DNA damage, immunosuppression, and angiogenesis, processes that ultimately support tumor progression.

## 11. Conclusions

In the TME, a variety of stresses impair canonical cap-dependent translation, so tumor cells exploit IRES-mediated, cap-independent translation to ensure the continued synthesis of key oncogenic and survival-related proteins. This review focuses on how tumors can escape the protein synthesis block, highlighting IMT as a crucial adaptive mechanism in tumor progression and therapy resistance. Despite their sequence diversity IRESs are generally highly structured and modulated, with conformations that provide specific binding platforms for ITAFs which promote ribosome recruitment independent of the 5′ cap.

The interplay between the IRES architecture and ITAFs is critical for translational control in response to stress; however, this aspect has remained understudied despite offering multiple therapeutic interventions. Indeed, recent preclinical studies have identified small molecules capable of selectively inhibiting IRES function. For example, silvestrol, a natural compound that acts on the eIF4A helicase, disrupts the unwinding of structured IRES regions [163], thereby impairing IRES-mediated translation of oncogenes. Similarly, riluzole has shown efficacy in suppressing IRES-dependent translation in glioblastoma models [164]. These findings provide experimental evidence that pharmacological targeting of IRES can limit tumor growth and sensitize tumor cells to apoptosis.

These results are rather modest and insufficient to confidently consider this type of therapeutic approach. As previously reported, each IRES may present a unique panel of interacting proteins, different from each other, related to the lack of common sequence or structural motifs. Therefore, the challenge posed by this approach is to identify a common denominator essential for the cap-independent translation mechanism, regardless of the structural heterogeneity of the IRES elements. Until then, studies to identify molecules capable of blocking the IMT of a group of IRESs, as well as their transport into the TME, are crucial [165]. These include nanoparticle-based carriers and tumor-targeted conjugates to improve selectivity and minimize systemic toxicity. Targeting the interaction between ITAF and IRES, via small molecules, antisense oligonucleotides, or RNA aptamers, represents a promising approach to selectively reduce oncogenic translation.

Engineering IRES-containing vectors for gene therapy allows for the coexpression of multiple therapeutic genes, enabling controlled and coordinated protein production. Such vectors hold the potential for synergistic cancer treatments by combining pro-apoptotic factors with immunomodulators [166]. Emerging technologies such as CRISPR-based RNA editing and antisense therapies may offer exciting opportunities to directly disrupt IRES RNA structures or modulate ITAF expression with high precision. Furthermore, integrating IRES-targeted treatments with conventional immunotherapy or chemotherapy may overcome resistance mechanisms in cancer cells.

In conclusion, comprehensive understanding of IRES structural biology, ITAF dynamics, and their context-dependent regulation within the TME will be essential to translate these insights into effective clinical interventions.

## Data Availability

Not applicable.
